# PP-ISEA: An Efficient Algorithm for High-Resolution Three-Dimensional Geometry Reconstruction of Space Targets Using Limited Inverse Synthetic Aperture Radar Images

**DOI:** 10.3390/s24113550

**Published:** 2024-05-31

**Authors:** Rundong Wang, Weigang Zhu, Chenxuan Li, Bakun Zhu, Hongfeng Pang

**Affiliations:** Space Engineering University, Beijing 101400, China; zifan_tao@hgd.edu.cn (R.W.); lccxmail@163.com (C.L.); zbk@hgd.edu.cn (B.Z.); panghongfeng@126.com (H.P.)

**Keywords:** three-dimensional (3D) geometry reconstruction, inverse synthetic aperture radar (ISAR) image sequence, energy accumulation, partitioned parallel

## Abstract

As the variety of space targets expands, two-dimensional (2D) ISAR images prove insufficient for target recognition, necessitating the extraction of three-dimensional (3D) information. The 3D geometry reconstruction method utilizing energy accumulation of ISAR image sequence (ISEA) facilitates superior reconstruction while circumventing the laborious steps associated with factorization methods. Nevertheless, ISEA’s neglect of valid information necessitates a high quantity of images and elongated operation times. This paper introduces a partitioned parallel 3D reconstruction method utilizing sorted-energy semi-accumulation with ISAR image sequences (PP-ISEA) to address these limitations. The PP-ISEA innovatively incorporates a two-step search pattern—coarse and fine—that enhances search efficiency and conserves computational resources. It introduces a novel objective function ‘sorted-energy semi-accumulation’ to discern genuine scatterers from spurious ones and establishes a redundant point exclusion module. Experiments on the scatterer model and simulated electromagnetic model demonstrate that the PP-ISEA reduces the minimum image requirement from ten to four for high-quality scatterer model reconstruction, thereby offering superior reconstruction quality in less time.

## 1. Introduction

Inverse Synthetic Aperture Radar (ISAR) has demonstrated its capability to detect non-cooperative targets with high precision and long range, irrespective of the time of day or night [1,2,3,4,5,6]. Researchers have capitalized on the broad bandwidth of the transmitted signal and the Doppler effect to achieve high resolution in both range and azimuth directions for ISAR imaging. However, ISAR imaging results present challenges such as geometric distortion, attitude sensitivity, and unknown projection planes. With the continuous increase in spacecraft targets in space [7,8] and the steady diversification of target types, the utilization of 2D information encounters significant limitations and struggles to satisfy the requirements of subsequent target identification. As a response to these challenges, techniques for 3D geometry reconstruction of space targets based on ISAR images have been extensively employed to enhance the feature extraction capability of ISAR images and to achieve a higher recognition rate.

Techniques for 3D geometry reconstruction predicated on ISAR images can be bifurcated into two categories, contingent upon the system distinction: multi-sensor-based ISAR image 3D geometry reconstruction [9,10,11,12,13,14,15,16,17] and single-sensor-based 3D geometry reconstruction [18,19,20,21,22,23,24,25,26,27,28,29]. Within the realm of 3D reconstruction employing multiple sensors, interferometric inverse synthetic aperture radar (InISAR) is the most prevalently utilized method. In this approach, a radar system with multiple sensors concurrently observes the target, given the relative positional differences of the sensors and the target, the received signals of each sensor exhibit unique wave path differences, resulting in variegated phases for each pixel in the image. These differences can be resolved through the principle of interferometry to procure the three-dimensional positional information [9,10,11]. Prior to the execution of interferometric 3D imaging, preliminary measures such as antenna configuration [12,13] and image registration [14,15,16] are requisite. Scholars have developed a suite of algorithms facilitating airborne and satellite-borne interferometric imaging, along with multi-static InISAR 3D imaging. It is worth mentioning that the recent method of 3D polarimetric InISAR imaging of non-cooperative targets [17] selects the optimal polarization combination that achieves the highest coherence and signal-to-noise (SNR) ratio, thereby enabling precise interferometric phase 3D imaging. Although interferometric imaging algorithm requires shorter observation time than single-sensor-based 3D geometry reconstruction algorithm, InSAR impose substantial prerequisites on the operational milieu, including a pronounced correlation among ISAR images from respective radars, stringent baseline-to-radar distance specifications, and intricate signal processing demands.

Single-sensor 3D geometry reconstruction necessitates prolonged observation and leverages the relative kinematics between target and radar to acquire an ISAR image sequence depicting various perspectives. Subsequent processing extracts the third-dimensional information. The seminal technique for 3D reconstruction from image sequences is the factorization method outlined in [18], originally conceived for optical images and later adapted to ISAR images as reported in [19]. The inherent sparsity of ISAR images compounds the challenges in 3D imaging, entailing a suite of preprocessing steps prior to reconstruction. These steps include feature point extraction [20,21,22,23] and trajectory association [24,25]. Now series of enhanced algorithms on feature point extraction and trajectory association have been proposed, [20,21,22,23] introducing advanced methodologies such as speeded-up robust feature (SURF) and SURF based on the theory of Geometric Algebra (GA-SURF). These innovations aim to facilitate more precise 3D reconstruction through the stabilization of feature extraction, supported by empirical experimentation. Additionally, research documented in [24,25] has refined trajectory association across diverse target motion models, enhancing the accuracy of associations for incomplete trajectories, thereby improving the quality of reconstruction outcomes. While the factorization method simplifies the hardware demands of radar systems and reduces associated costs, the algorithmic process involved remains complex and prone to instability. Consequently, the accurate extraction and matching of scattering points persist as significant challenges, impacting the effectiveness of 3D reconstruction efforts. Furthermore, radar systems based on single sensors can exploit target rotation characteristics to achieve imaging in specific scenarios [26,27,28], and recently, the RaNeRF method has been proposed, employing deep learning techniques for high-quality reconstruction of target images [29].

In order to address the above issues, Lei Liu proposed ISEA in [30], which leverages the projection principle of ISAR imaging plane. This approach recognizes that the projection energy accumulated by genuine scatterers per frame significantly surpasses that of other locations. Utilizing the particle swarm optimization (PSO) algorithm, ISEA facilitates target reconstruction. This technique achieves enhanced reconstruction quality through a streamlined process. However, ISEA necessitates the computation of the imaging plane of space targets, contingent on the radar’s line of sight (LOS) orientation and attitude of the space targets, thereby restricting its applicability to tri-axially stable targets in space. Subsequently, Zuobang Zhou tackled the limitation of ISEA’s inability to reconstruct three-axis unstable targets [31,32,33]. Zhou’s method employs the quantum-behaved particle swarm optimization (QPSO) algorithm to estimate the rotational motion parameters of slow rotating space targets (SRSTs), facilitating successful reconstruction. Furthermore, an extended factorization framework (EFF) has been introduced to accommodate targets with uncertain motion patterns, enhancing the versatility of ISEA and improving its applicability in real-world scenarios. However, achieving high-accuracy reconstruction of simple scattering points with ISEA still demands over ten ISAR images [30], the inherently limited search algorithm of ISEA leading to unnecessary computational resource consumption and significant time expenditure.

The imperfection of ISEA can be attributed to the loss of critical information and the inherent limitations of the PSO algorithm in addressing complex challenges. In response, this paper aims to enhance the reconstruction efficacy by undertaking innovative work grounded on the aforementioned insights.

1. Considering the limitations of the PSO algorithm when dealing with highly complex reconstruction tasks, this study proposes dividing the search area into sub-blocks and adopting a two-step search method: a preliminary coarse search followed by a more precise fine search. This approach not only simplifies the complexity of the problem, thereby conserving computational resources, but also facilitates parallel processing, enhancing the efficiency of 3D reconstruction.
2. ISEA overlooks certain valuable information, resulting in suboptimal reconstruction outcomes with limited imagery. Consequently, this paper aims to attain stable reconstruction results using fewer images by extracting the projection information from each frame. It introduces a novel objective function designed to more effectively differentiate between genuine and spurious scattering points.
3. Addressing the issue of outliers in reconstruction, this paper presents a redundant point exclusion module that precisely eliminates outliers based on defined statistical characteristics, retaining only high-quality reconstructed points. This approach ensures the achievement of high-precision reconstruction.

The remainder of the paper is organized as follows: Section 2 constructs imaging geometry and expounds the module proposed herein. Subsequently, Section 3 executes empirical analyses on both scattering point and simulated electromagnetic computer-aided design (CAD) models. Finally, conclusive remarks are drawn in Section 4.

## 2. Materials and Methods

### 2.1. Imaging Scene and Signal Model

Establishing the imaging scene and signal model forms the foundation of imaging processing. Whether it is 2D or 3D imaging, determining the imaging scene is the initial and essential step. Only then can the radar echo be simulated, the ISAR image generated, and ultimately the 3D geometry of the target reconstructed.

**Figure 1:**
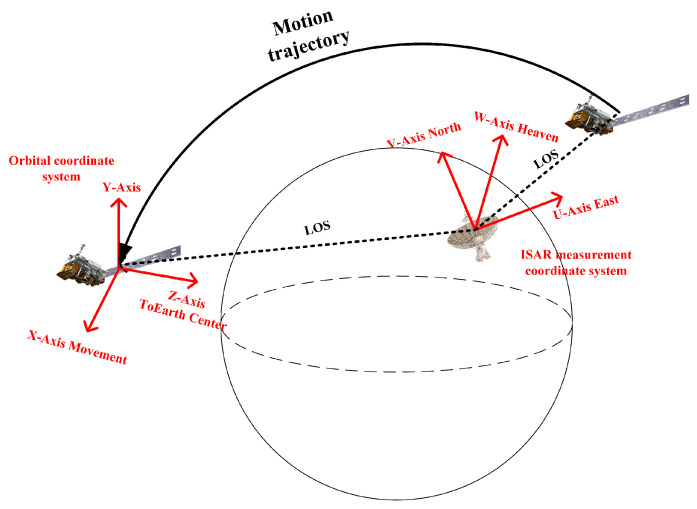
Radar imaging scene.

Figure 1 depicts the imaging scenario of this study, wherein a ground-based radar continuously emits coherent electromagnetic waves towards the space target. The target orbits the Earth according to its trajectory.

In order to achieve high-resolution imaging, radars generally emit broadband signals, and this paper takes the emitted signal $S_{t}$ as an example

(1) $$S_{t}(t)=exp(j2\pi ft)$$

**Figure 2:**
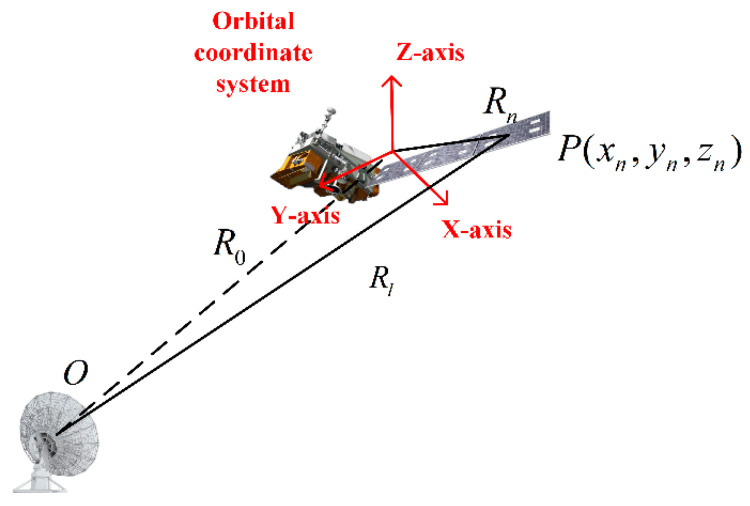
Schematic of radar and satellite positions.

The relative position of the space target to the radar is shown in Figure 2, the signal $S_{p}(t)$ reflected back from point P in space is as follows:

(2) $$S_{p}(t)=\rho (x_{n},y_{n},z_{n})exp\{j2\pi f[t-\frac{2r_{l}(t)}{c}]\}$$

where $\rho (x_{n},y_{n},z_{n})$ is the reflectivity density of the scatterer P at $\left(x_{n},y_{n},z_{n}\right)$, and $r_{l}(t)$ is the distance between the radar and the scatterer as a function of time. $S_{p}(t)$ is a function of the reflected signal at a single point, and for the whole object the echo signal should be an integral of the multiple scattering points, so the target echo $S_{r}(t)$ is as follows:

(3) $$S_{r}(t)=∭\rho (x_{n},y_{n},z_{n})exp\{j2\pi f[t-\frac{2r_{l}(t)}{c}]\}dxdydz$$

adjust to the fundamental frequency:

(4) $$S_{r}(t)=∭\rho (x_{n},y_{n},z_{n})exp\{-j4\pi f\frac{r_{l}(t)}{c}\}dxdydz$$

As shown in Figure 2, since $r_{0}(t)>>r_{n}$, the radar-target distance $r_{l}$ can be approximated as the projection of the sum of the target scattering point vector and the radar position vector in the LOS direction, i.e.,

(5) $$r_{l}(t)={\left(R_{n}(t)-R_{0}(t)\right)}^{T}\times l$$

where, l is the LOS direction, $R_{0}(t)$ is the distance vector between the target’s center point and the radar, $R_{n}(t)$ is the distance vector between the target’s center and the target scattering point. The LOS direction and $R_{0}(t)$ in the ISAR measurement coordinate system (RMCS) are as follows:

(6) $$l=\left[\begin{array}{c} cos\varphi (t)cos\theta (t)\\ cos\varphi (t)sin\theta (t)\\ sin\varphi (t) \end{array}\right]$$

(7) $$R_{0}(t)=\left[\begin{array}{c} r_{0}(t)cos\varphi (t)cos\theta (t)\\ r_{0}(t)cos\varphi (t)sin\theta (t)\\ r_{0}(t)sin\varphi (t) \end{array}\right]$$

where φ(t) and θ(t) are the pitch angle and azimuth angle of the LOS in RMCS, $r_{0}(t)$ denotes the distance between the target’s center and the radar.

To represent l, $R_{0}(t)$, and $R_{n}(t)$ in the same coordinate system, a transformation matrix is used to convert l and $R_{0}(t)$ from RMCS to OCS:

(8) $$l_{OCS}=T_{OR}\cdot l=\left[\begin{array}{c} -cos\varphi^{\prime }(t)cos\theta^{\prime }(t)\\ -\cos\varphi^{\prime }(t)sin\theta^{\prime }(t)\\ -sin\varphi^{\prime }(t) \end{array}\right]$$

(9) $$R_{0}^{OCS}(t)=T_{OR}\cdot R_{0}(t)=\left[\begin{array}{c} r_{0}cos\varphi^{\prime }(t)cos\theta^{\prime }(t)\\ r_{0}cos\varphi^{\prime }(t)sin\theta^{\prime }(t)\\ r_{0}sin\varphi^{\prime }(t) \end{array}\right]$$

(10) $$R_{n}(t)=H_{motion}(t)\cdot \left[\begin{array}{c} x_{n}\\ y_{n}\\ z_{n} \end{array}\right]=\left[\begin{array}{c} x_{n}(t)\\ y_{n}(t)\\ z_{n}(t) \end{array}\right]$$

where $\varphi^{\prime }(t)$ and $\theta^{\prime }(t)$ represent the pitch and azimuth angles of the LOS in OCS, $T_{OR}$ refers to the transformation matrix from RMCS to OCS. $l_{OCS}$ and $R_{0}^{OCS}(t)$ respectively denote the LOS and $R_{0}(t)$ in the OCS. $R_{n}(t)$ represents the coordinates of a specific scattering point of the target in the OCS. If the target is three-axis unstable, $H_{motion}(t)$ denotes its motion characteristics. If the object is a three-axis stable target, then the position of the scattering point is determinate, i.e., $R_{n}$= ${\left[\begin{array}{ccc} x_{n} & y_{n} & z_{n} \end{array}\right]}^{T}$.

Substituting the above vectors into Equation (5), yields the radar’s distance $r_{l}(t)$ from the scattering point:

(11) $$r_{l}(t)=r_{0}(t)-x_{n}(t)\cos\varphi^{\prime }(t)\cos\theta^{\prime }(t)-y_{n}(t)\cos\varphi^{\prime }(t)\sin\theta^{\prime }(t)-z_{n}(t)\sin\varphi^{\prime }(t)$$

Substituting Equation (11) into Equation (4), the received signal can be obtained as follows:

(12) $$\begin{array}{l} S_{r}(t)=\exp[-j4\pi f\frac{r_{0}(t)}{c}]\\ ∭\rho (x_{n},y_{n},z_{n})exp\{j2\pi [x_{n}(t)f_{x}(t)+y_{n}(t)f_{y}(t)+z_{n}(t)f_{z}(t)]\}dx_{n}dy_{n}dz_{n} \end{array}$$

where, $f_{x}(t)=\frac{2f\cos\varphi^{\prime }(t)\cos\theta^{\prime }(t)}{c}$, $f_{y}(t)=\frac{2f\cos\varphi^{\prime }(t)\sin\theta^{\prime }(t)}{c}$, $f_{z}(t)=\frac{2f\sin\varphi^{\prime }(t)}{c}$.

In summary, we can deduce the echo reflected by the target in the 3D space under uncertain circumstances, in which $r_{0}(t)$ is a function of time, which can represent the target’s motion of translation; $\varphi^{\prime }(t)$ and $\theta^{\prime }(t)$ represent the LOS’s pitch angle and azimuth angle variation under the OCS, and $R_{n}(t)=\left[\begin{array}{ccc} x_{n}(t) & y_{n}(t) & z_{n}(t) \end{array}\right]$ is also function of time, reflecting the target’s rotation situation.

**Figure 3:**
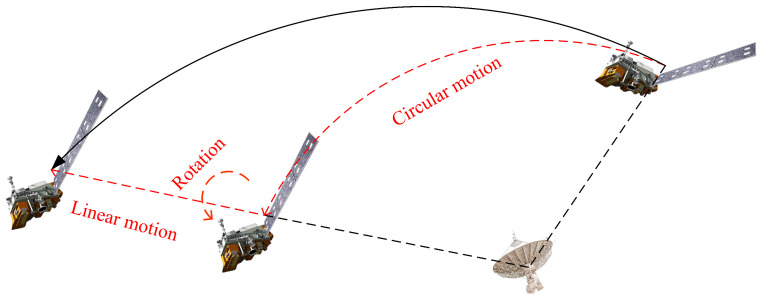
Schematic of the relative motion between the radar and the satellite.

Figure 3 illustrates the relative motion between the radar and the satellite, where the satellite’s orbit comprises circumferential and rotational motion as well as translational movement. The circular motion of the satellite is inconsequential to imaging, while variations in LOS and the satellite’s unknown rotation contribute to Doppler resolution in imaging. Moreover, translational motion towards the end of the trajectory may interfere with satellite imaging, necessitating compensation for accurate imaging. Owing to high-frequency band and wide bandwidth, direct application of the range-Doppler (RD) algorithm for imaging may lead to migration through range cells (MTRC) and resultant defocusing [34,35]. To address this issue, the polar formatting algorithm (PFA) can correct MTRC, enhance image quality [36,37], and facilitate improved reconstruction outcomes. 

### 2.2. 3D Geometry Reconstruction Method Utilizing ISAR Image Sequence 

#### 2.2.1. Flowchart

**Figure 4:**
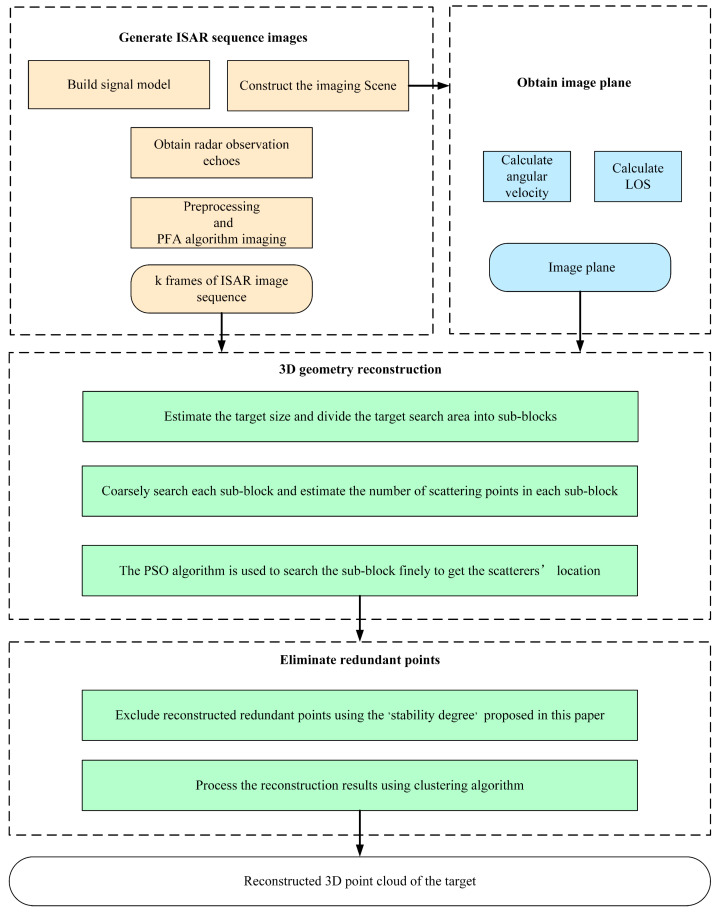
Flowchart of the proposed method in this paper.

Figure 4 depicts the workflow for implementing the method proposed in this study, containing four procedures. Initially, the imaging scene is constructed to simulate echo signals, following this, compensations for translational effects are applied, and PFA is utilized to acquire a high-quality ISAR image sequence—fundamental for 3D reconstruction. The second step involves computing the projection relationship and defining the imaging plane in accordance with the radar scenario. This commences with a preliminary estimation of the target’s dimensions using the ISAR images, subsequent establishment of an initial search area, and division of target area into smaller sub-blocks. The number of scattering points contained in each sub-block can be predicted based on the projection information of the sub-blocks on the ISAR images, thereby completing a coarse search of the target area. The fine search then utilizes the PSO algorithm, informed by the coarse search, to meticulously locate scatter points through an enhanced objective function ‘sorted-energy semi-accumulation’, thus facilitating the reconstruction of scatter points for each sub-block. However, due to the emergence of certain outliers during the search, this necessitates the implementation of the fourth step: the filtration of preliminary reconstruction results via a redundant point exclusion module developed in this study, which employs a unique statistical characteristic to eliminate superfluous scatterer points. Subsequent employment of clustering algorithms amalgamates these filtered results, culminating in a comprehensive reconstructed scatter point model.

#### 2.2.2. Principle of the Imaging Plane

For 3D ISAR imaging, its essence is the 2D projection of the 3D model on the ISAR imaging plane, the two dimensions of the ISAR imaging plane are the range dimension and the Doppler dimension; the ISAR imaging plane can be determined through these two dimensions.

Derived from Section 2.1, for a space target exhibiting three-axis stability, the radar-target distance is as follows:

(13) $$\begin{array}{l} r_{l}(t)=r_{0}(t)-x_{n}cos\varphi^{\prime }(t)cos\theta^{\prime }(t)\;\;\;\;\;\;\;\;\;\;\;\;\;\;\;\;\;\;\;\;\;\;\;\;\;\;\;\;\;\;\;\;\;\;\;\;\;\;\\ \;\;\;\;\;\;\;\;\;\;\;\;\;\;\;\;\;\;\;\;\;\;\;\;\;\;\;\;\;\;\;\;\;\;\;\;\;\;-y_{n}cos\varphi^{\prime }(t)sin\theta^{\prime }(t)-z_{n}sin\varphi^{\prime }(t) \end{array}$$

The Doppler frequency resulting from the instantaneous distance variation between the radar and the target in the LOS direction can be calculated as follows:

(14) $$f_{n}(t)=-\frac{2}{\lambda }\cdot \frac{\partial r_{l}(t)}{\partial t}$$

here λ is the wavelength of the emitted electromagnetic wave, and substituting Equation (13) into Equation (14) is able to obtain the following equation:

(15) $$\begin{array}{l} f_{n}(t)=-\frac{2}{\lambda }\cdot \{x_{n}\left[-\frac{\partial \varphi^{\prime }(t)}{\partial t}cos\theta^{\prime }(t)sin\varphi^{\prime }(t)-\frac{\partial \theta^{\prime }(t)}{\partial t}sin\theta^{\prime }(t)cos\varphi^{\prime }(t)\right]\\ +y_{n}\left[-\frac{\partial \varphi^{\prime }(t)}{\partial t}sin\theta^{\prime }(t)sin\varphi^{\prime }(t)+\frac{\partial \theta^{\prime }(t)}{\partial t}cos\theta^{\prime }(t)cos\varphi^{\prime }(t)\right]+z_{n}\frac{\partial \varphi (t)}{\partial t}cos\varphi (t)\} \end{array}$$

By this point, we have the instantaneous projected distance of the radar’s LOS and the magnitude of the instantaneous Doppler frequency, in effect, we take these two variables as the two coordinate axes of the ISAR imaging, and the projection is given by the following:

(16) $$\left[\begin{array}{l} r_{n}^{k}\\ f_{n}^{k} \end{array}\right]=\left[\begin{array}{l} {\left(\rho_{r}^{k}\right)}^{T}\\ {\left(\rho_{a}^{k}\right)}^{T} \end{array}\right]\cdot R_{n}$$

where *k* denotes the index for the number of image frames, *n* signifies the scattering point index, $r_{n}^{k}$ and $f_{n}^{k}$ denote the projected coordinates of the *n*th scattering point in the *k*th frame along the range and cross-range axes, respectively. Additionally, $\rho_{r}^{k}$ and $\rho_{a}^{k}$ represent the unit vectors corresponding to the range and cross-range directions. The unit vectors in the range and azimuth directions can be obtained by combining Equations (13), (15) and (16):

(17) $$\rho_{r}^{k}=\left[\begin{array}{c} -cos\varphi^{\prime }(t)cos\theta^{\prime }(t)\\ -cos\varphi^{\prime }(t)sin\theta^{\prime }(t)\\ -sin\varphi^{\prime }(t) \end{array}\right]$$

(18) $$\rho_{a}^{k}=\frac{2}{\lambda }\left[\begin{array}{c} -\omega_{\varphi }(t)cos\theta^{\prime }(t)sin\varphi^{\prime }(t)-\omega_{\theta }(t)sin\theta^{\prime }(t)cos\varphi^{\prime }(t)\\ -\omega_{\varphi }(t)sin\theta^{\prime }(t)sin\varphi^{\prime }(t)+\omega_{\theta }(t)cos\theta^{\prime }(t)cos\varphi^{\prime }(t)\\ \omega_{\varphi }(t)cos\varphi^{\prime }(t) \end{array}\right]$$

where $\omega_{\varphi }(t)=\frac{\partial \varphi^{\prime }(t)}{\partial t}$, $\omega_{\theta }(t)=\frac{\partial \theta^{\prime }(t)}{\partial t}$ are the partial derivatives of the pitch and azimuth angles, respectively, i.e., the angular velocities in both directions which connects the 3D coordinate system with the range–Doppler coordinate system. So the ISAR images used in this method can be in the range–Doppler coordinate system, eliminating the need for scaling to range-cross-range coordinates.

**Figure 5:**
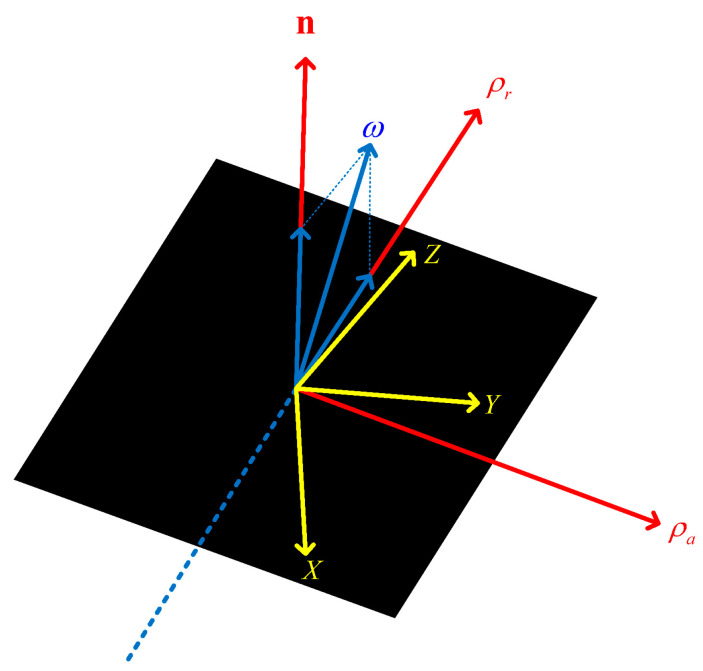
ISAR imaging plane.

In Figure 5, the yellow coordinate system illustrates the target body coordinate system (OCS), with the black plane representing the projected ISAR imaging plane. The blue dashed and solid lines denote the radar’s LOS direction and the angular velocity vector, respectively. The red coordinate system denotes the radar imaging coordinate system. The range direction, corresponding to $\rho_{r}^{k}$, is defined by the radar’s transmission and reception of electromagnetic waves to and from the target, whereas the cross-range direction, associated with $\rho_{a}^{k}$ and defined by Equation (18), pertains to the target’s angular velocity.

In summary, the imaging plane is ascertainable through the LOS and the angular velocity, thereby each 2D ISAR image can be determined from the 3D target model. Conversely, if the ISAR image sequence of a target is known, the information of the ISAR image sequence and the LOS vector information can be used to reconstruct the target model inversely according to the principle of ISAR plane.

#### 2.2.3. Partitioned Coarse Search

This study introduces a two-step search strategy comprising a preliminary coarse search followed by a fine search, wherein the target area is initially segmented into smaller sub-blocks. The coarse search evaluates the energy magnitude within each sub-block, furnishing foundational data for the fine search. This approach facilitates intensified scatter point investigations in higher energy zones, moderates search efforts in lower energy areas, and eliminates searches in regions virtually devoid of energy.

The partitioned coarse search methodology offers several advantages:

1. It streamlines computational resource utilization by curtailing unnecessary expenditure, thereby enhancing search efficiency through strategic allocation.
2. By decomposing a complex optimization challenge characterized by multiple peak values across a vast expanse into simpler, manageable problems, the method addresses the PSO algorithm’s limitations in accurately identifying true scatterers amidst intricate scenarios. This refinement will yield more precise solutions and elevates the success rate of target reconstruction.
3. The segmentation of the target domain into numerous sub-blocks transforms a complicated iterative ordeal into several discrete, simpler iterations. This segmentation satisfies the conditions for parallel processing, significantly accelerating the search process.

Nevertheless, the execution of the coarse search necessitates a criterion to evaluate the energy magnitude within these sub-blocks. This requires the anticipation of scatter point density within sub-blocks to dictate the extent of the search effort for scatterers accordingly. If a sub-block is posited to encompass n scattering points, then its 2D projection on any ISAR image frame will include at minimum the projections of those n entities. Additionally, due to the way of calculating the projection matrix of the 3D space and the 2D image, a non-bijective relationship exists between spatial coordinates and their corresponding image locations. Consequently, other scattering points may coexist within this projected region, implying that the projection energy of this spatial sub-block within a specific ISAR image frame should be greater than or equal to the cumulative energy of its inherent scattering points. Based on this rationale, we have adopted the following computational rule shown below as the predicted value:

(19) $$N_{opt}=\min\left\lceil \frac{E_{i}^{k}\times N}{E_{total}^{k}}\right\rceil ,k=1,2,\cdots ,N_{f},i=1,2,\cdots ,N_{areas}$$

⌈ ⌉ denotes upward rounding, *k* signifies the image sequence frame index, *i* represents the region index, *N* constitutes the estimated number of scattering points included in the model, $E_{total}^{k}$ denotes the aggregate energy of all scattering points across the *k*th frame of the image, and $E_{i}^{k}$ signifies the collective energy within the ith projection region in the *k*th frame.

#### 2.2.4. Fine Search in Sub−Blocks

Subsequent to partition, the PSO algorithm is harnessed to conduct targeted searches within each sub−block, predicated on the prognosticated scatter point density. The PSO methodology unfolds as follows [30,38]:

- In the first step: n probing particles are instantiated within 3D space, both initial positions and velocities of particles are determined randomly. According to the projection rule described in Section 2.2.2, the objective function can be constructed based on the energy information of the search point projected on each ISAR image, (for the specific way of constructing the objective function, please refer to Section 2.2.5), and the initial objective function value of each particle is subsequently calculated.
- In the second step: the objective function value corresponding to the initially set search particles is calculated as the initial personal best objective function value for each particle, and the position is taken as the personal best position, and the particle position with the highest objective function value is also selected as the global best position.
- In the third step: according to the rules of Equations (20) and (21), update the velocity and position of the individual particles:

(20) $$v_{i}^{k+1}=\omega v_{i}^{k}+c_{1}r_{1}(p_{i,pbest}^{k}-x_{i}^{k})+c_{2}r_{2}(p_{gbest}^{k}-x_{i}^{k})$$

(21) $$x_{i}^{k+1}=x_{i}^{k}+v_{i}^{k+1}$$

where *k* denotes the number of iterations, *i* denotes the particle number, $v_{i}^{k+1}$ denotes the velocity of the *i*th search particle after *k* + 1 iterations, ω denotes the weight of the next iteration’s velocity by the current velocity, $v_{i}^{k}$ denotes the velocity of the *i*th search particle after k iterations, $p_{i,pbest}^{k}$ denotes the optimal position of the *i*th particle at the *k*th iteration, and $p_{gbest}^{k}$ denotes the global optimal position at the *k*th iteration, $c_{1}$, $c_{2}$ denote the weights of the velocity vector of the next iteration of the search example influenced by the current personal best and global best position, respectively. $r_{1}$, $r_{2}$ are two random numbers that can influence the velocity direction in terms of both the personal best and the global best, respectively.

- In the fourth step: iterative update, after the completion of the velocity and position update, it should be based on the calculation of the objective function value of each particle. Update the individual best position of each particle as well as the global best position, judge whether to satisfy the termination conditions, if yes, then complete the cycle, otherwise return to the third step, and update the new position of each particle again.
- Finally: set the loop end condition, and the algorithm iterates to be able to obtain the final scattering points for the search.

#### 2.2.5. Objective Function Improvement

In the ISEA algorithm, the quality of reconstruction largely depends on solving the optimization problem, with the objective function as its pivotal element. Specifically, the ISEA’s objective function aggregates the projected energy on every ISAR image, predicated on the assertion that true scatterer locations manifest higher energy accumulations compared to non−scatterer locations. But at certain spatial locations where no true scattering point exists, its projected position may also fall at high energy locations in the image; calculations based on the original objective function are likely to produce erroneous reconstruction points. This suggests that the original objective function may not capture all the nuanced information present in the sequence of images. For a more accurate identification of true scattering points in 3D space, we need a revised perspective: the true scatterer exists at the location where the projected point on each frame of the ISAR images has high energy and is not equivalent to having the highest projected energy sum at that location. The original ISEA’s objective function is slightly one−sided and may lead to cases of erroneous reconstruction, thereby adopting a new metric to define a more robust objective function will improve the reconstruction outcome. The original objective function of ISEA is as follows:

(22) $$E=\sum_{k=1}^{N_{f}}I_{k}(p\cdot \rho_{r}^{k}+\frac{M_{r}}{2},p\cdot \rho_{a}^{k}+\frac{M_{c}}{2}),k=1,2,\cdots ,N_{f}$$

where $I_{k}$ refers to the *k*th frame of the ISAR sequence image, $p=(x_{s},y_{s},z_{s})$ is the search point position vector, $\rho_{r}^{k}$ and $\rho_{a}^{k}$ refer to the range direction and cross−range direction in the *k*th frame image, and $M_{r}$, $M_{c}$ refer to the number of cells in the range and cross−range directions of the ISAR image, respectively.

This paper introduces a novel objective function, termed “sorted−energy semi−accumulation”, according to the necessary condition for the existence of a true scattering point at a spatial location. This method entails calculating the projected energy values at a specified location for each frame, followed by ranking these values from highest to lowest. A true scattering point is characterized by superior energy values across all image frames, including the number of frames at the bottom of the ordering, compared to areas devoid of scatterers.

(23) $$E_{new}={\left[\begin{array}{c} I_{1}(p\cdot \rho_{r}^{1}+\frac{M_{r}}{2},p\cdot \rho_{a}^{1}+\frac{M_{c}}{2})\\ I_{2}(p\cdot \rho_{r}^{2}+\frac{M_{r}}{2},p\cdot \rho_{a}^{2}+\frac{M_{c}}{2})\\ \begin{array}{l} \vdots \\ I_{N_{f}}(p\cdot \rho_{r}^{N_{f}}+\frac{M_{r}}{2},p\cdot \rho_{a}^{N_{f}}+\frac{M_{c}}{2}) \end{array} \end{array}\right]}_{sort}^{T}\cdot I_{E},k=1,2,\cdots ,N_{f}$$

where $N_{f}$ is the frame number of ISAR image sequence, and ${\left[\;\;\;\right]}_{sort}$ denotes sorting the elements from smallest to largest.$I_{E}={\left[\begin{array}{ccccc} 1 & 1 & \cdots & 0 & 0 \end{array}\right]}^{T}$, is a 1 by k matrix with 1 s and 0 s each occupying k/2 of the elements, the first half of the elements being ones and the second half being zeros.

The innovative objective function effectively discriminates against locations that exhibit high projection values in only a limited frame, thereby facilitating the reconstruction of the target’s three−dimensional structure with enhanced precision.

#### 2.2.6. Redundancy Point Elimination

In the application of the PSO algorithm for the meticulous localization of scattering points within sub−blocks, it necessitates conducting searches predicated on the anticipated density of scattering points as determined by a preliminary, coarse search. To avoid overlooking genuine scatter points in fine search, the predicted number of scatter points in coarse search will inevitably exceed the actual scatter points, thereby inevitably leading to the generation of redundant scatterers.

To address the challenge of eliminating these spurious scattering points. This study draws inspiration from the concept of coefficient of variation (Cv), and introduces novel metrics aimed at expunging surplus scatterers engendered by the partitioned coarse search. As delineated in Equation (24), the Cv denotes the ratio of standard deviation to the mean, serving as a foundational component of our methodology.

(24) $$Cv(X)=\frac{\sqrt{Var(X)}}{Mean(X)}$$

where X is the sample, *Var* denotes the variance of the sample, and *Mean* denotes averaging over the sample.

The verification of a point as an authentic scatterer hinges on consistent high energy values across all frames, it is necessary to exclude situations where the total energy is high due to high projection energy in a few frames of images. Spatial coordinates without genuine scatterers may manifest higher energy projections in certain frames than actual scatterers, yet the energy in other frames is almost zero. True scattering points are characterized by two principal attributes: elevated energy and high stability—aspects inadequately captured by the original objective function.

By leveraging the mean and standard deviation of energy projections across frames, we introduce a “stability degree” metric to quantify the authenticity of a scattering point’s location, thus incorporating a comprehensive evaluation of both high energy and stability into the assessment process:

(25) $$St(E_{p})=\frac{Mean(E_{p})}{Std(E_{p})+\frac{1}{N_{p}}\sum_{p=1}^{N_{p}}Std(E_{p})}$$

where $E_{p}$ refers to the vector of $1\times N_{f}$ projection values of a position on each frame of the image, $N_{p}$ is the number of positions of the search result, and $N_{f}$ is the number of frames for which the reconstruction is performed.

To mitigate the inclusion of outliers, this study calculates the ‘stability degree’ of currently identified scattering points, selecting scattering point with higher stability degree, retaining 1.2 times the anticipated number N of scattering points. In this approach, there is still the persistence of superfluous scattering points—equivalent to 0.2 times N—distributed around the authentic scattering points, necessitating further action for accurate reconstruction. The extant scattering points, encompassing both genuine and adjacent lower energy scatterers, are then subjected to consolidation. The k−means clustering algorithm is employed to amalgamate the information pertaining to these scattering points. By clustering 1.2 times the number of scattering points into predetermined clusters, the centroids of these clusters are delineated as the ultimate reconstructed scattering point models, thereby refining the precision of the spatial localization.

### 2.3. Performance Analysis

#### 2.3.1. Reconstruction Success Rate [26]

In order to measure the reconstruction effectiveness of the scattering point model, it is necessary to establish the ‘reconstruction success rate’ index, and first of all, match the reconstructed scattering point with the ideal scattering points, and calculate whether there exists a real scattering point within a certain range of the reconstructed point. If there is a true scattering point, this means the matching is successful, and if there is no true scattering point within the range, then the reconstruction of the reconstructed point fails, and there is no true scattering point that matches this reconstructed scattering point.

The reconstruction success rate is defined as the ratio of whether there exists a reconstructed scattering point matching each real scattering point, as shown in Equation (26):

(26) $$R_{r}=\frac{N_{matched}}{N_{real}}\times 100\%$$

where $N_{matched}$ represents the number of real scattering points that were successfully matched and $N_{real}$ represents the number of all real scattering points.

#### 2.3.2. Root−Mean−Square Error (RMSE)

Calculating the root−mean−square error between the coordinates of the reconstructed scattering points and the coordinate of the true scattering points can reflect the accuracy of 3D geometric reconstruction.

(27) $$E_{RMSE}=\sqrt{\frac{1}{N_{real}}\sum_{i=1}^{N_{real}}{\left(P_{i}^{opt}-P_{i}^{real}\right)}^{T}(P_{i}^{opt}-P_{i}^{real})}$$

where $P_{i}^{opt}$ denotes the coordinates of the ith reconstructed point, and $P_{i}^{real}$ denotes the position coordinates of the corresponding ith real scattering point. The reconstruction rmse can reflect the reconstruction effect; the better the reconstruction effect, the closer the two positions are, and the smaller the reconstruction rmse calculated according to the position coordinates.

#### 2.3.3. Reconstruction Accuracy and Integrity [28]

In order to accurately assess the reconstruction effectiveness of complex scattering point data of 3D models as well as simulated electromagnetic data, the reconstruction success rate index of simpler scattering point models is no longer applicable, so reconstruction accuracy and integrity are established based on the idea of differentiation to assess the complex models.

Firstly, the target area is gridded according to the idea of differential, and the length of the grid is taken as 1/5 of the shortest side of the target, and the target is placed completely within the grid area. After this, it is possible to determine the distribution of the point cloud in each mesh, and the distribution of the real target model and the reconstructed target model in each mesh is calculated separately to obtain the mesh in which the two models are present. We obtain $C_{real}$ as the mesh contained in the real model. $C_{reconstruct}$ as the mesh contained in the reconstructed model, and $C_{effective}$ as the mesh contained in both, i.e., the portion that was successfully reconstructed.

The reconstruction accuracy is the ratio of the successfully reconstructed grid $C_{effective}$ to the grid $C_{reconstruct}$ occupied by the overall reconstructed points:

(28) $$\eta =\frac{C_{effective}}{C_{reconstruct}}\times 100\%$$

The integrity of the reconstruction is defined as the ratio of the successfully reconstructed mesh $C_{effective}$ to the mesh $C_{real}$ where the real model is located:

(29) $$\varsigma =\frac{C_{effective}}{C_{real}}\times 100\%$$

## 3. Results

To authenticate the efficacy of the proposed algorithm in terms of accuracy and robustness, this section conducts a validation of the novel modules delineated within this paper. Experimental evaluations are performed on an array of models, encompassing simple and complex scattering point models, as well as simulated electromagnetic CAD models. The assessments compare the outcomes utilizing both the conventional ISEA methodology and the PP−ISEA approach, thereby offering a comprehensive appraisal of proposed modules.

### 3.1. Simple Scattering Point Model Experiment

To prove that the method proposed in this paper is superior to the original ISEA method and has practical significance, in this section, based on the simple scattering point model, the proposed module of this paper is first validated and finally the overall reconstruction effect is compared with the original ISEA method.

#### 3.1.1. Simple Scattering Point Model Imaging Scene Parameters

**Figure 6:**
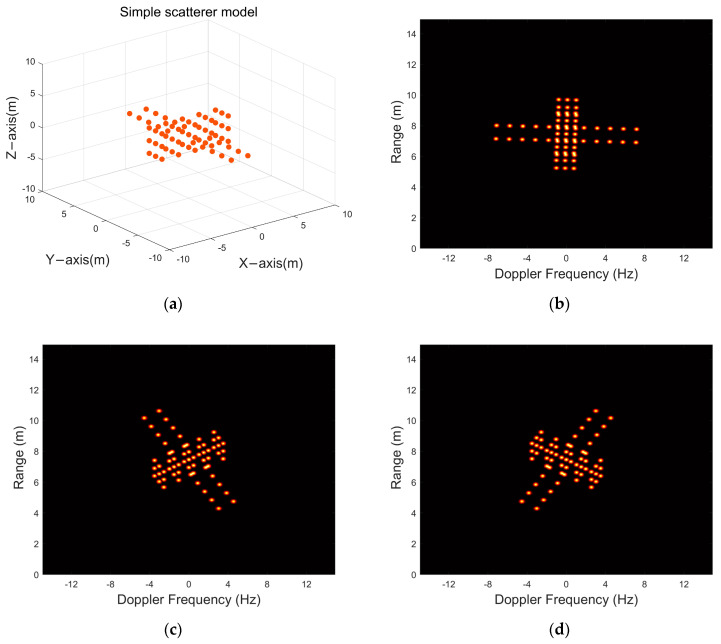
Simple scatterer model and its ISAR image: (**a**) simple scatterer model, (**b**), (**c**) and (**d**) are the 1st, 8th, and 16th frames of the ISAR image sequence, respectively.

Figure 6a shows the 65−point satellite model established in this paper, and this satellite is a three−axis stabilized target that moves in space according to the satellite orbit. Ground−based radar observes it with coherent accumulation, and the specific parameters are shown below:

Long−duration observations of space targets using the parameters shown in Table 1. are capable of yielding thousands of echoes, to achieve imaging of the target. Firstly, the echoes need to be divided into sub−apertures, and next, if the RD algorithm is used for imaging, the phenomenon of MTRC occurs, and high resolution ISAR images can be obtained using PFA correction. The ISAR images of the satellite at different frames are shown in Figure 6b–d. To prove PP−ISEA can achieve high−quality reconstruction with fewer ISAR images than ISEA, The ISAR image quality in this paper matches that of [30], with a Signal−to−Noise Ratio (SNR) of 45 dB. Because the complete long time coherent accumulated echoes of the target cannot be obtained in all cases, this paper takes the number of received radar echoes as a variable to study and aims to achieve high quality reconstruction even with a small number of echoes.

#### 3.1.2. Verification of Objective Function Modification

In this study, the objective function underlying the conventional ISEA method has been enhanced to more effectively discriminate between authentic and spurious scattering points. This section undertakes a comparative analysis of the original and revised objective functions, focusing on their performance across two distinct spatial coordinates, to ascertain the superiority of the novel objective function.

**Figure 7:**
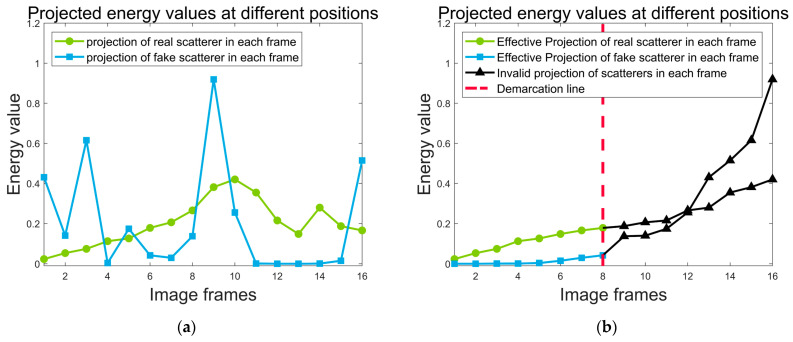
Projected energy for different frames: (**a**) energy projection of two different positions in different frames. (**b**) Energy projection of two different positions in different frames after sorting.

Figure 7 delineates the energy projection maps for two spatial locations across various frames within a sixteen−frame image reconstruction scenario. The erroneous reconstruction points are indicated by blue polyline, and the authentic scattering points are depicted through green polylines, facilitating an analysis of their respective energy projection values across individual image frames. A genuine scattering point is characterized by consistently projecting values across all frames. Contrastingly, the blue polylines exhibit significant disparities in energy projections, with pronounced peaks in certain frames but markedly low values in others. This inconsistency suggests the absence of a true scattering point at the locations corresponding to the blue polylines. Conversely, while the peak values of the green polylines may not surpass those of the blue ones, its uniformly high projection values across all frames substantiate the presence of a legitimate scattering point. This analysis verifies the augmented precision of the revised objective function in segregating real scattering points from artefactual ones.

In ISEA, the differentiation between the existence or non−existence of scattering points at a specific spatial position is initially determined by the computation of the original objective function, which constitutes the accumulation of projected energies. Interestingly, the energy accumulation at true scattering points is 3.1896, slightly lower than that of fake scattering points, which is 3.2787. The revised objective function, however, selects the eight lowest sums of projected energy from the sixteen−frame image as its reference for target reconstruction. Under the revised computational rules, the objective function at true scattering point is 0.8812, whereas at spurious scattering point, it is 0.0936, the value associated with the true scattering point location is nearly an order of magnitude greater than that corresponding to the false scattering point location. This objective function significantly enhances the capacity to discern the presence of scattering points at the two spatial locations, as substantiated by the data presented in Table 2. 

#### 3.1.3. Validation of Coarse Search Scattering Point Count Prediction

**Figure 8:**
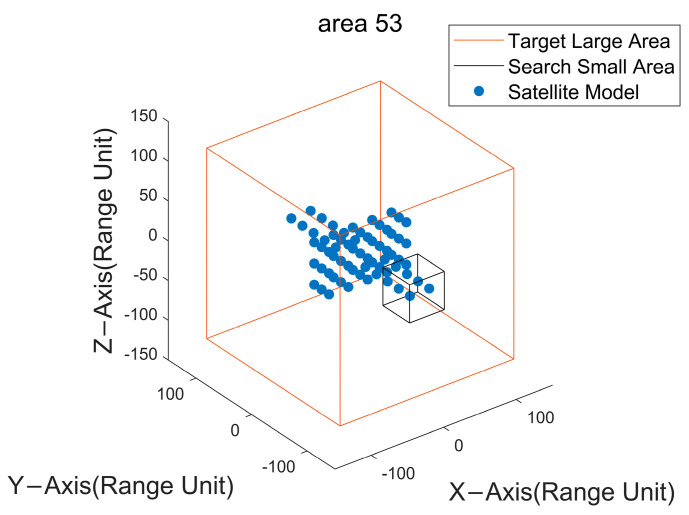
Schematic of area 53 with satellite model.

**Figure 9:**
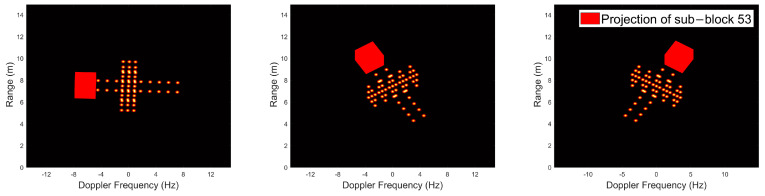
Projections of area 53 onto the frames.

The reconstruction methodology proposed in this paper encompasses two steps: an initial coarse search followed by a fine search. The preliminary stage, coarse search, necessitates the assessment of the quantity of genuine scattering points within each delineated sub−block. This entails dividing the search region—spanning length, width, and height—into equitably sized partitions. The positioning of the fifty−third spatial partition is depicted in Figure 8, with subsequent mappings of its projection across individual frames illustrated in Figure 9.

**Figure 10:**
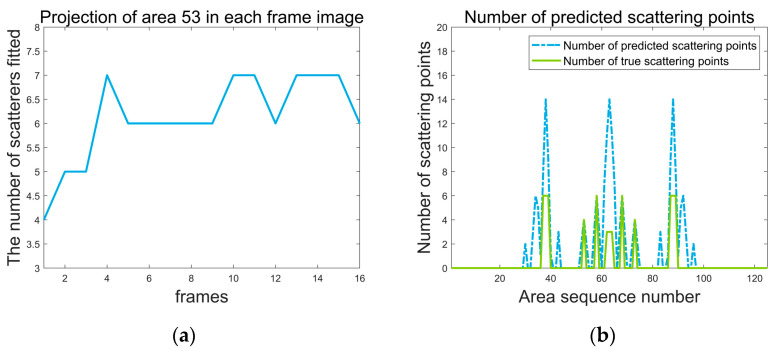
(**a**) Prediction of region 53 containing scattering points using each frame alone. (**b**) Prediction results for regions containing scattering points using PP−ISEA.

Figure 10a represents the prognosticated count of scattering points, which is founded on the ratio of projected energy by the specific region within each frame relative to the aggregate energy embodied across the entirety of the image.

(30) $$N_{i}^{k}=\left\lceil \frac{E_{i}^{k}\times N}{E_{total}^{k}}\right\rceil ,k=1,2,\cdots ,N_{f},i=1,2,\cdots ,N_{areas}$$

where *i* is the region index, *k* is the frame index, *N* is the total number of scattering points contained in the model, $E_{i}^{k}$ is the energy contained in region *i* of the *k*th frame, and $E_{total}^{k}$ is all the energy contained in the *k*th frame of the ISAR image.

Utilizing data from individual frames to forecast the quantity of scattering points within area 53, which comprises four authentic scattering points, Figure 10a illustrates that the inaugural frame exhibits the minimal energy level. This minimal energy projection accurately predicts the area’s content of four scattering points. Conversely, in subsequent frames, the fitted point count exceeds four, attributed to the incorporation of energy from additional scattering points. Thus, employing the least amount of energy as a predictive measure for the fitted number emerges as the most logical strategy:

(31) $$N_{est}^{i}=\min\left[\left\lceil \frac{E_{i}^{1}\times N}{E_{total}^{k}}\right\rceil ,\left\lceil \frac{E_{i}^{2}\times N}{E_{total}^{k}}\right\rceil ,\cdots ,\left\lceil \frac{E_{i}^{N_{f}}\times N}{E_{total}^{k}}\right\rceil \right],i=1,2,\cdots ,N_{areas}$$

This section encompasses experimental validation of the theoretical framework presented in Section 2.2.3, conducting a coarse search across each sub−block to estimate scattering point numbers, with outcomes displayed in Figure 10b.

The green solid line delineating the actual count of scattering points per sub−block, and the blue dashed line indicating the coarse search’s predictive estimations. Remarkably, the blue dashed line consistently remains slightly higher or equal than the green solid line, indicating that within each sub−block, the predicted number of scattering points exceeds the actual number to a certain extent. This methodology ensures no genuine scattering points are overlooked while concurrently avoiding an excessive increment in search iterations per region. These fitting results furnish a foundational basis for the ensuing fine search phase.

#### 3.1.4. Processing of Redundant Points

**Figure 11:**
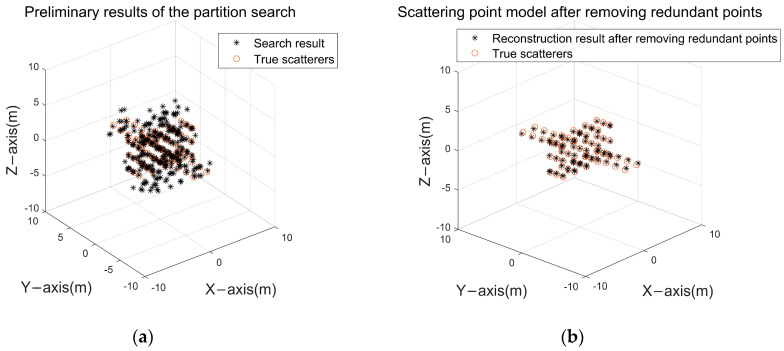
Redundancy point exclusion: (**a**) preliminary result of the partition search; (**b**) scattering point model after removing redundant points.

**Figure 12:**
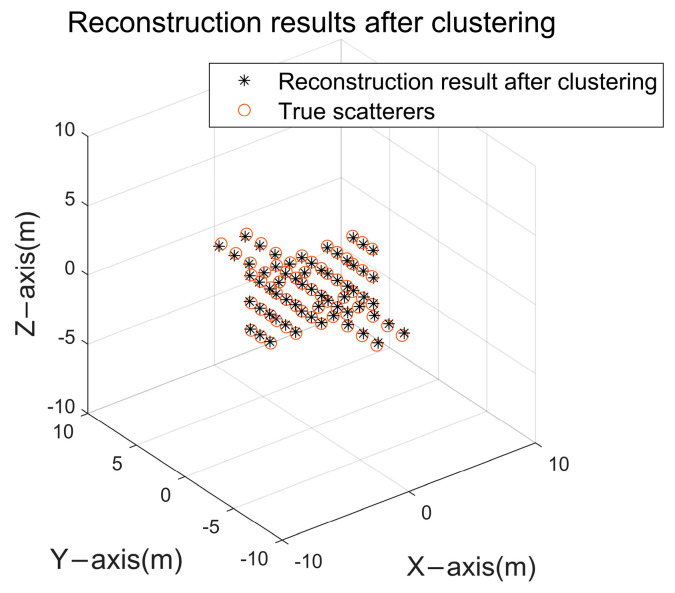
Final redundancy point elimination results.

The redundant point exclusion module proposed in this paper is validated using a simple scattering point model, and the partitioned search results are shown in Figure 11a, which contains a large number of redundant points, and reconstruction results after excluding outliers are shown in Figure 11b according to the index “stability degree” proposed in this paper:

In Figure 11b, although a large number of redundant points with small projection energy are excluded, there still contains a small number of redundant points around the real scattering points, so a clustering operation can be carried out to fuse the redundant points dispersed in the real scattering points, and the result is shown in Figure 12.

After excluding redundant points, the reconstruction success rate of the scattering point model reaches 100%. A clear comparison between Figure 12 and Figure 11 demonstrates the effectiveness of the redundant point exclusion module in handling redundant points after partitioned search, yielding the expected outcomes.

#### 3.1.5. Comparison of the Effect on Simple Scattering Point Reconstruction

**Figure 13:**
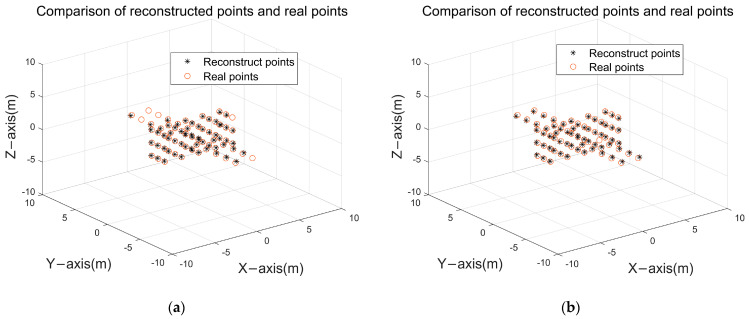
Comparison of reconstruction results: (**a**) ISEA method reconstruction results; (**b**) reconstruction results utilizing PP−ISEA.

To demonstrate the superiority of the PP−ISEA methodology, this study contrasts the reconstruction outcomes of a 65−point satellite target employing the conventional ISEA technique against those achieved via PP−ISEA. The experimental design subdivided the target echo into 16 sub−apertures, generating 16 ISAR image frames for the purpose of reconstruction. Figure 13a delineates the results obtained through the application of the ISEA method, while Figure 13b presents the reconstruction outcomes facilitated by PP-ISEA.

The comparative analysis indicates that PP−ISEA improves the successful reconstruction rate of satellites compared to ISEA. While ISEA achieved a reconstruction accuracy of 90.77%, PP−ISEA increases this figure to 98.46%, as shown in Table 3. Concurrently, the RMSE has undergone a marginal shift from 0.0884 to 0.0945, indicating no substantial diminution. Furthermore, the execution duration for the algorithm is dramatically condensed from the initial 3960 s to a mere 601 s.

With the improvement in this paper, the new method is supposed to reduce the requirement on the number of radar echoes and achieve a higher reconstruction success rate using fewer pictures. To assess this proposition, subsequent experiments were designed, utilizing the quantity of image frames as the independent variable to appraise the efficacy of diverse reconstruction methodologies.

**Figure 14:**
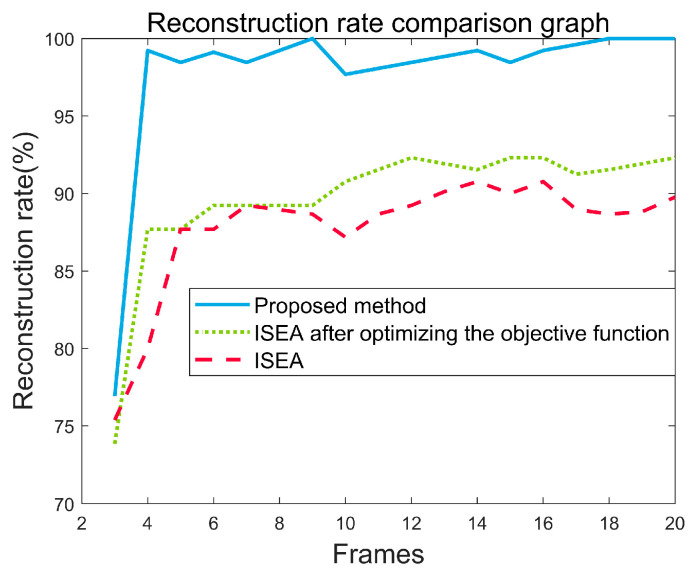
Trend plot of reconstruction success rate for different methods.

In Figure 14, reconstructions of the simplified satellite model were performed using varying number of frames. Extensive experimentation revealed that adjusting the objective function of ISEA can slightly improve the success rate of target reconstruction. However, it fails to achieve high−quality reconstruction with a limited number of ISAR images. Experimental results on PP−ISEA illustrate that even with just four image frames, PP−ISEA achieves an impressive reconstruction rate of 99.23%, significantly surpassing the traditional ISEA approach. 

### 3.2. Complex Scattering Point Model Validation

To further validate the effectiveness as well as the robustness of the method proposed in this paper, this section extends the reconstruction algorithm to the reconstruction of complex scattering point models. 

The experiments in this section are based on point cloud models from the Hubble Space Telescope (HST) as well as the Aqua satellite to generate target ISAR images for reconstruction.

**Figure 15:**
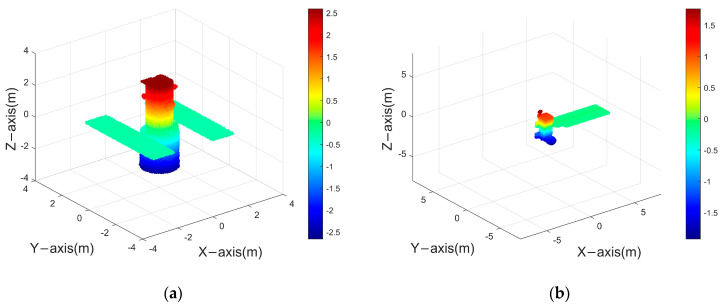
Satellite model: (**a**) HST (**b**) Aqua.

**Figure 16:**
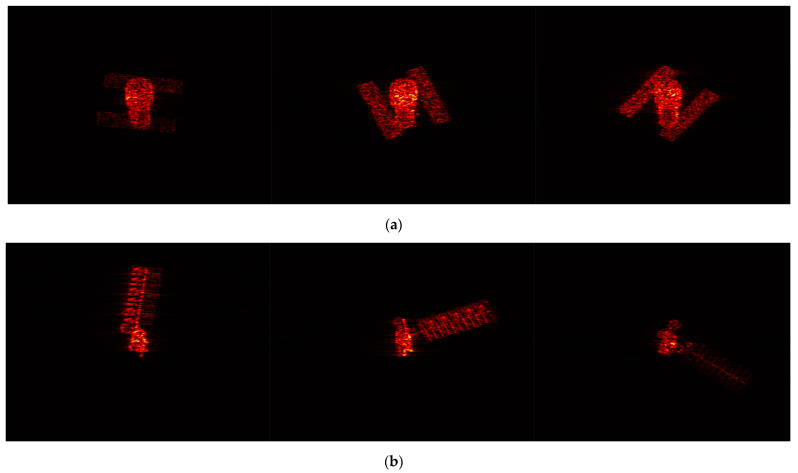
ISAR image sequence of space targets: (**a**) ISAR image sequence of HST; (**b**) ISAR image sequence of Aqua.

Figure 15 presents the point cloud model of a space target, with varying colors denoting different altitude levels. Utilizing the parameters listed in Table 4, coherent radar echoes were generated to observe the space target. Subsequently, a set of 30 ISAR images was produced. Figure 16 displays selected frames—specifically, the 1st, 15th, and 30th ISAR images derived from the Hubble Space Telescope (HST) and the Aqua satellite.

**Figure 17:**
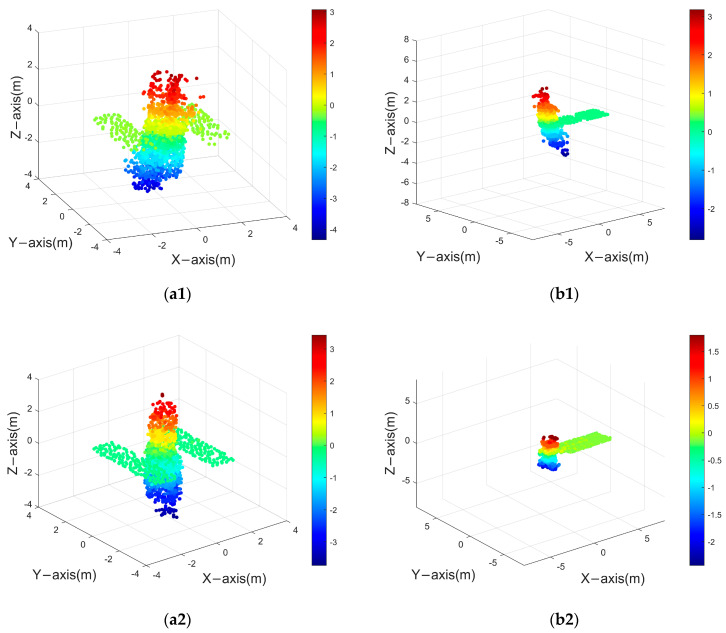
Space target reconstruction result: (**a1**) reconstruction result of HST using the ISEA method; (**a2**) reconstruction result of HST using PP−ISEA; (**b1**) reconstruction result of Aqua using the ISEA method; and (**b2**) reconstruction result of Aqua using PP−ISEA.

The reconstruction of the target was undertaken employing both the ISEA methodology and the PP−ISEA technique. Figure 17 illustrates the outcomes of this reconstruction process, whereas Table 5 details the reconstruction accuracy and completeness achieved by each method.

From the experiments, ISEA’s reconstruction result of complex scattering point models using only 30−frame images does not work well. Conversely, PP−ISEA employing an identical quantum of images, demonstrates enhanced efficiency with reduced computational time. Moreover, it achieves superior reconstruction accuracy and completeness in comparison to the ISEA approach for the reconstruction of complex scattering point models. The reconstruction accuracy increased by at least 10%, while the reconstruction integrity also improved by at least 6%, exhibiting strong superiority.

### 3.3. Electromagnetic Data Validation

**Figure 18:**
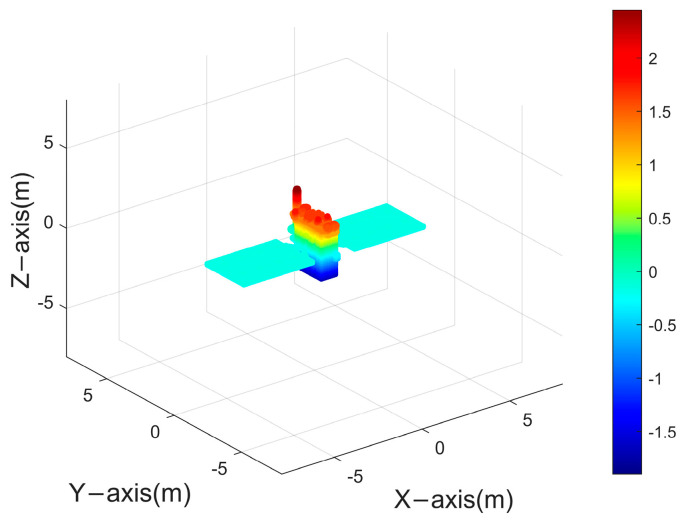
Point cloud model of space target.

**Figure 19:**
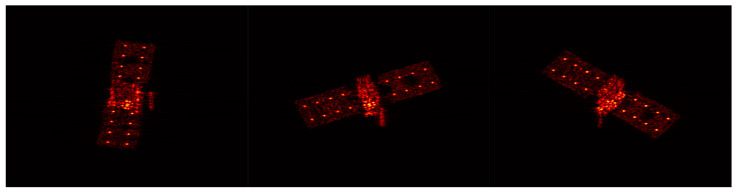
ISAR image of space target.

Electromagnetic simulation represents the closest approximation to measured data. Within this study, electromagnetic simulation software has been employed to generate the ISAR images of a space target. Figure 18 showcases the satellite’s point cloud model, which serves to facilitate the generation of a 30−frame ISAR image sequence. Observation of the target was conducted through azimuth angles ranging from 0° to 137.5°, under a fixed pitch angle of 30°. A selection of these ISAR image frames is depicted in Figure 19.

**Figure 20:**
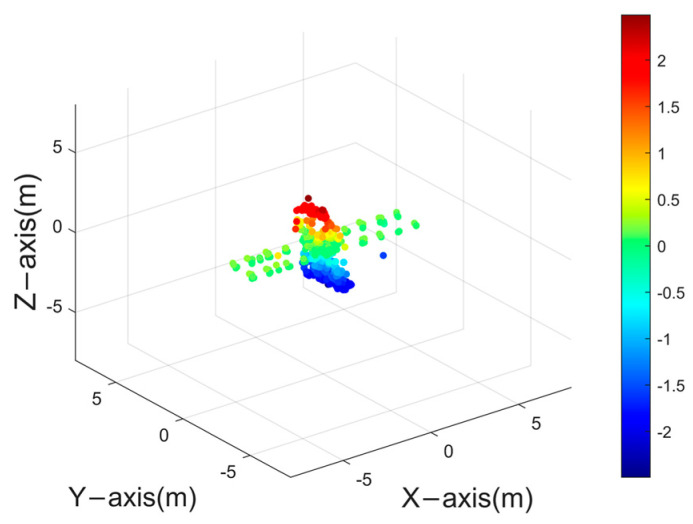
Reconstruction results for electromagnetic simulation of space targets using PP−ISEA.

Figure 20 presents the outcome of the reconstruction process, achieving an accuracy of 92.64% and an integrity of 84.29%. The reconstruction accuracy meets the requirements, but there is still room for further improvement in reconstruction integrity. This is attributed to the presence of more complex scenarios in both electromagnetic simulation images and real ISAR images. Due to variations in scattering coefficients of the same scatter point under different viewing angles, varying degrees of anisotropy in scatter point magnitudes will be observed in ISAR images.

## 4. Discussion and Conclusions

This paper enhances the ISEA to yield superior reconstructions. The ISEA’s original objective function, ‘energy accumulation’, omits critical data during the processing of ISAR image sequences. To address this, the proposed method, dubbed PP−ISEA, introduces a refined objective function, ‘sorted−energy semi−accumulation’, which better exploits accessible information. Furthermore, ISEA’s optimization algorithm struggles with complex reconstruction scenarios, prompting PP−ISEA to introduce a two−step search strategy—comprising both coarse and fine searches—to streamline the optimization challenge. Additionally, leveraging the coefficient of variation, PP−ISEA devises a novel metric for the accurate exclusion of erroneously identified scattering points. Section 3 of the paper presents comprehensive experimental validations that underscore the efficacy of PP−ISEA. Specifically, the novel objective function more effectively discriminates between authentic and spurious scattering points. The innovative search pattern facilitates judicious allocation of computational resources and enhances reconstruction efficiency via parallel processing techniques. The addition of a redundant point exclusion module effectively filters outlier scattering points. Consequently, PP−ISEA harnesses the potential of extant image sequence data and mitigates the detrimental impacts of search algorithm constraints on reconstruction quality. For the simple scattering point model, PP−ISEA has the capability to reduce the required number of images for reconstruction from 10 to 4, achieving an impressive reconstruction success rate of 99.23%. In the case of the complex point model, utilizing 30 images, PP-ISEA demonstrates a reconstruction accuracy exceeding ISEA by 10% and a reconstruction completeness surpassing it by over 6%. Future research avenues include integrating PP−ISEA’s methodologies with existing ISEA tailored for dynamic space objects, aiming to achieve high−fidelity reconstructions in real−world scenarios.

## Figures and Tables

**Table 1:** Simple scattering point model parameters.

Parameters	Value
Frequency	15 Ghz
Bandwidth	1 Ghz
Pitch angle	$30^{\circ }$
Azimuth angle	$0^{\circ }-120^{\circ }$

**Table 2:** Effect of different objective functions.

	Real Scatterer	Fake Scatterer
Energy accumulation value	3.1896	3.2787
Sorted−energy semi−accumulation value	0.8812	0.0936

**Table 3:** Reconstruction effects of different methods using 16−frame images.

	ISEA	Proposed Method
Reconstruction success rate (%)	90.77	98.46
Root−mean−square error (m)	0.0884	0.0945
Reconstruction time (s)	3960	601

**Table 4:** Table of parameters for the complex scattering point model.

Parameters	Value
Duration of observation	$137.5^{\circ }$
Number of ISAR image	30
Frequency	15 Ghz
Bandwidth	4 Ghz
Pitch angle	$30^{\circ }$
Azimuth angle	$0^{\circ }–120^{\circ }$

**Table 5:** Reconstruction results of complex scattering point models using different methods.

	HST	Aqua
Accuracy using ISEA	78.84%	81.37%
Integrity using ISEA	84.13%	87.46%
Accuracy using proposed method	91.95%	90.91%
Integrity using proposed method	93.54%	93.24%
Time consumed by ISEA	82,417 s	67,253 s
Time consumed by proposed method	10,625 s	8627 s

## Data Availability

Data are contained within the article.
